# Metformin sensitizes therapeutic agents and improves outcome in pre-clinical and clinical diffuse large B-cell lymphoma

**DOI:** 10.1186/s40170-020-00213-w

**Published:** 2020-07-06

**Authors:** Anil R. Singh, Juan J. Gu, Qunling Zhang, Pallawi Torka, Suchitra Sundaram, Cory Mavis, Francisco J. Hernandez-Ilizaliturri

**Affiliations:** 1 Texas Oncology, Texas, USA; 2 Department of Medicine, Roswell Park Comprehensive Cancer Center, Buffalo, USA; 3 Department Immunology, Roswell Park Comprehensive Cancer Center, Buffalo, USA; 4 Department of Medical Oncology Fudan University Shanghai Cancer Center, Shanghai, People’s Republic of China

**Keywords:** Metformin, Rituximab-chemotherapy resistance, Lymphoma, Glucose metabolism

## Abstract

**Background:**

The treatment of diffuse large B-cell lymphoma (DLBCL) is limited by the development of resistance to therapy, and there is a need to develop novel therapeutic strategies for relapsed and refractory aggressive lymphoma. Metformin is an oral agent for type 2 diabetes that has been shown to decrease cancer risk and lower mortality in other types of cancer.

**Methods:**

We performed a retrospective analysis of the RPCCC database looking at patients with DLBCL treated with front-line chemotherapy. We also performed pre-clinical studies looking at the effect of metformin on cell viability, cell number, Ki67, ATP production, apoptosis, ROS production, mitochondrial membrane potential, cell cycle, effect with chemotherapeutic agents, and rituximab. Finally, we studied mouse models to see the anti-tumor effect of metformin.

**Results:**

Among diabetic patients, metformin use was associated with improved progression-free survival (PFS) and overall survival (OS) compared to diabetic patients not on metformin. Our pre-clinical studies showed metformin is itself capable of anti-tumor effects and causes cell cycle arrest in the G1 phase. Metformin induces apoptosis, ROS production, and increased mitochondrial membrane permeability. Metformin exhibited additive/synergistic effects when combined with traditional chemotherapy or rituximab in vitro. In vivo, metformin in combination with rituximab showed improved survival compared with rituximab monotherapy.

**Conclusions:**

Our retrospective analysis showed that metformin with front-line chemotherapy in diabetic patients resulted in improved PFS and OS. Our pre-clinical studies demonstrate metformin has potential to re-sensitize resistant lymphoma to the chemo-immunotherapy and allow us to develop a hypothesis as to its activity in DLBCL.

## Introduction

The need to improve upon therapeutic approaches for relapsed/refractory diffuse large B-cell lymphoma (DLBCL) patients was highlighted by the results of the prospective multicenter phase III Collaborative Trial in Relapsed Aggressive Lymphoma (CORAL) study. DLBCL patients previously treated with rituximab (R) in combination with standard doses of cyclophosphamide, doxorubicin, vincristine, and prednisone (R+CHOP) had only a 34% event-free survival (EFS) after R-based salvage immunochemotherapy followed by high-dose chemotherapy and autologous stem cell transplant (HDC-ASCT) [1, 2]. Scientific efforts must be focused in defining the resistance pathways developed by lymphoma cells and integrate therapeutic strategies to overcome them. To this end, we developed several rituximab-resistant cell lines (RRCL) and found that the acquirement of rituximab resistance leads to resistance to multiple chemotherapy agents [3, 4]. Perhaps related to the acquirement of rituximab-chemotherapy resistance observed in our RRCL, we found a de-regulation of apoptosis, cell cycle progression, and glucose metabolism [4–7]. Cancer cells alter their mitochondrial potential (i.e., apoptotic threshold) to resist the cytotoxic effects from the host immune-surveillance cells and/or the toxic effects of therapeutic interventions. As a consequence, the cellular metabolism shifts from aerobic to anaerobic glycolysis in order to generate adenosine triphosphate (ATP) (Warburg effect) [8, 9]. In an attempt to maintain adequate ATP levels and meet their energy requirement, those cancer cells are required to maintain a higher glucose uptake rate [10, 11].

Metformin has been widely prescribed to type II diabetics since 1950. The exact mechanism(s) by which metformin lowers blood glucose levels is poorly defined. However, several hypotheses had been postulated: (1) inhibition of hepatic gluco-neogenesis and (2) reduction of insulin resistance enhancing glucose uptake in skeletal muscle [12–14]. In addition, metformin appears to exhibit anti-cancer properties. Metformin at supra-physiological (mM range) doses is an agonist of the adenosine monophosphate-activated protein kinase (AMPK) that plays a pivotal role in cellular metabolism and B-cell development. It has been demonstrated that AMPK inhibits the mammalian target of rapamycin (mTOR), partially explaining its anti-tumor effects. Another known anti-cancer effect of metformin is through the inhibition of complex 1 in the mitochondrial electron transport chain leading to ATP reduction [15–17]. Epidemiological studies have demonstrated that the use of metformin in diabetic patients is associated with a decrease in the incidence of cancer or lower cancer-related mortality [18–23].

In DLBCL, there is conflictive data on the impact of the use of metformin in DLBCL outcomes. A retrospective study from Dr. Solomon group and a prospective study from Dr. Zhao group found that metformin use prolonged the survival of DLBCL, whereas another retrospective study conducted by Dr. Cerhan group failed to demonstrate a positive clinical benefit in the same lymphoma subtype [24–26]. Several clinical trials combined metformin with either sirolimus or temsirolimus with R-CHOP or DA-EPOCH-R are current evaluating in NHL [27].

In our current work, we present the results of a retrospective study conducted at our Institute. In it, we found that DLBCL diabetic patients who used metformin during first-line chemo-immunotherapy had a significantly improved progression-free survival (PFS) and overall survival (OS) compared to non-diabetic or diabetic DLBCL patients that used other glucose-lowering agent(s). Our findings suggested a therapeutic role of metformin in DLBCL. Subsequently, using both, in vitro and in vivo lymphoma pre-clinical models as well as primary clinical samples obtained through our lymphoma clinical program, we identified metformin as an effective therapeutic drug against B-cell NHL. To our knowledge, this is the first report that demonstrates metformin’s anti-cancer properties in resistant B-cell lymphoma. Our findings support the combination of metformin with other chemotherapeutic agents as relatively inexpensive and potentially effective approach to reverse the drug-resistance in B-cell lymphoma.

## Methods

### Differences in the clinical outcomes following first-line chemo-immunotherapy in diabetic and non-diabetic DLBCL patients.

Using the RPCCC tumor registry, we identified 264 DLBCL patients treated with rituximab and anthracycline-based therapy between 1997 and 2013. The cohort of patients included 49 diabetic patients. Demographic, clinical, pharmacological, and pathological characteristics were recorded (age, sex, DLBCL subtype according to the Han’s algorithm [28], stage at diagnosis, international prognostic index [IPI] risk category, treatment type, and whether or not they received radiation as consolidation to their first line of therapy). The use of metformin or other glucose lowering agents was recorded for each diabetic patient. Differences in response rate, PFS, and OS were evaluated between non-diabetic, diabetic on metformin, or diabetic on other glucose lowering agent DLBCL patients.

### Cell lines and primary patient samples

A panel of rituximab-sensitive (RSCL) or RRCL cell lines was cultured in RPMI1640 for the experiments as previously described [3, 4]. Primary patient samples from biopsy specimens were procured under Roswell Park Comprehensive Cancer Center Review Board protocols I42804 and I42904. Primary neoplastic B-cells were isolated from pre-treatment biopsy tissue obtained from 16 patients with previously untreated (*N* = 9) or relapsed/refractory (*N* = 7) NHL receiving therapy at RPCCC as previously described [29].

### In vitro effect of metformin on DLBCL cell viability, cell number, ATP, and Ki67

RRCL or RSCL were exposed in vitro to escalating doses of metformin for 24, 48, or 72 h. Cells were plated at a cell density of 0.5 × 10^6^ cells/ml. Cell proliferation was determined as the change in Presto blue (ThermoFisher, CA) reduction by living cells and measured using a FluoroScan Ascent LF (Thermo Fisher Scientific, Barrington, IL). The half maximal inhibition concentration (IC_50_) of metformin was calculated using the Graph Pad Prism Software version 6.04 (graph Pad Software, La Jolla, CA). Cell number in each condition was counted by Trypan blue exclusion. Changes in ATP production were determined using the Cell Titer-Glo Luminescent Viability Assay reagent (Promega). Experiments were done in triplicates and the percentage of ATP was assessed and normalized to controls. Ki-67 was stained using a FITC labeled mousse anti-human Ki-67 for 1 h and evaluated by flow cytometry analysis.

### Effects of metformin on apoptosis induction, radical oxygen species (ROS) production, and changes mitochondrial potential

Lymphoma cells were incubated at a cell density of 0.5 × 10^6^/mL in complete media containing DMSO or metformin (16 mM). After 48 h, cells were stained with Annexin V and PI in Annexin binding buffer (Thermos Fisher, Grand Island, NY). Following staining, 10,000 events were collected on a FACScan (Becton Dickinson). Data were analyzed using the FCS express software (De Novo Software, Los Angeles, CA), and differences in apoptosis induction were compared using paired *t* tests in the SPSS 14.0 software (SPSS, Inc.).

RSCL and RRCL were exposed to DMSO or metformin (16 mM) for 48 h. Subsequently, cells were re-suspended in 0.5 ml of PBS containing 5 μmol/l of dihydrorhodamine 123 (Invitrogen) and incubated at 37 °C for 30 min in the dark. ROS was determined by flow cytometry analysis. To determine changes in the mitochondrial potential, lymphoma cell lines were exposed to metformin (16 mM) for 48 h, and 1 × 10^6^ cells were incubated in DiOC6 (Thermofisher) at 37 °C for 30 min. The dose of DiOC6 used (20 nM) is within the ranges suggested by standard protocols. Scientists had doses ranging between 10 and 20 nM [30, 31]. We used FCCP treatment as a positive control. Cells were then washed and re-suspended in PBS and data collected and analyzed via flow cytometry.

### Effects of metformin on the cell cycle of RSCL and RRCL

RSCL and RRCL were exposed to metformin (8 or 16 mM) for 48 h. Cells were then washed with PBS and fixed with 70% ethanol at – 20 °C, incubated with 100 μg/ml RNase for 30 min (Sigma-Aldrich), and stained with 50 ug/ml PI. DNA content was determined by flow cytometry.

### Changes in the expression of several cell cycle proteins in RSCL or RRCL after exposure to metformin

RSCL and RRCL cells were exposed to metformin (8 mM) for 48 h, and changes in key regulators of the cell cycle pathway (C-Myc, PCNA, E2F and CDK2, (Cell Signaling Technologies, MA)) were evaluated using specific primary and secondary monoclonal/polyclonal antibodies by Western blot.

### Effects of metformin on transcription of C-Myc and PCNA

Changes in C-Myc and PCNA expression were analyzed with a quantitative real-time PCR using TaqMan primers and probes according to the manufacturer’s directions (Applied Biosystems). Briefly, after exposure to metformin (8 or 16 mM) for 48 h, total RNA was extracted and converted in complementary deoxyribonucleic acid (DNA) from RSCL and RRCL using the TaqMan Gene Expression cell-to-CT kit (Life Technologies) on an ABI-7500HT (Applied Bio systems). Ct values were determined using the SDS v 2.2 software (Applied Biosystems) and compared using the ΔΔCt method.

### Effect of metformin on complement-mediated cytotoxicity (CMC) and antibody-dependent cellular cytotoxicity (ADCC)

RSCL or RRCL were exposed to metformin (4, 8, or 16 mM) or DMSO for 48 h. Subsequently, 2 × 10^6^ viable cells were labeled with ^51^Cr at 37 °C, 5% CO_2_ for 2 h. ^51^Cr-labeled RSCL or RRCL were then placed in 96-well plates at a cell concentration of 1 × 10^5^ cells/well (CMC assay) or 1 × 10^4^ cells/well (ADCC assay). Cells were then exposed to rituximab or isotype (10 μg/ml) and human serum (CMC, 1:4 dilution) or PBMCs (ADCC, 40:1 effector: target ratio) for 6 h at 37 °C and 5% CO_2_. ^51^Cr release was measured as previous described [3].

### Effects of metformin on rituximab activity in vivo

For the in vivo experiments, 6–8-week-old severe compromised immunodeficiency (SCID) mice were utilized. SCID mice were inoculated on day zero with 10 × 10^6^ Raji cells through tail vein injection. After 72 h (to allow tumor engraftment), the animals were then divided into four cohorts. The first cohort (group A) was used as control and the animals did not receive any treatment. Group B consisted of animals treated with rituximab at 10 mg/kg on day +3, +7, +10, +14. Groups C were treated with metformin 2 μg/ml in drinking water until the experiment finished. Groups D was treated with combination treatment of both rituximab at 10 mg/kg and metformin 2 μg/ml in drinking water. The end point of the study was survival defined as the time to development of limb paralysis. Animals that reached the end point or survived after 3 months of observation were sacrificed by cervical dislocation. The experiments were repeated on three separate occasions.

## Results

### Patient characteristics

A total of 264 DLBCL patients were included in the retrospective analysis. All patients had DLBCL and received either R-CHOP, rituximab in combination with dose-adjusted etoposide, prednisone, vincristine, cyclophosphamide, and doxorubicin (R-DA-EPOCH) or R-CHOP-like chemotherapy (Table 1). A total of 215 patients were found to be non-diabetic and 49 patients were diabetic. Among diabetic patients, 21 were not on metformin, while 28 were using metformin (may or may not have been using other agents). Patients included were closely matched with regard to median age, sex, DLBCL subtype, initial stage, and IPI risk category at the time of diagnosis. The histologic subtype was closely matched among groups and was evenly divided among GCB, non-GCB, and unknown. Most patients presented with advanced stage disease (III or IV). There was a difference in the percentage of patients receiving R-DA-EPOCH in the non-diabetic patients (10.2%) compared to diabetic patients (0% not on metformin vs. 3.6% on metformin). There was also a difference in the rate at which radiation treatment was received among non-diabetic and diabetic patients on metformin (31.6% and 32.1% respectively) compared to diabetic patients not on metformin (10.0%). There was also a difference in the rate at which radiation treatment was received among non-diabetic and diabetic patients on metformin (31.6% and 32.1% respectively) compared to diabetic patients not on metformin (10.0%). There was a statistically significant difference between the non-diabetic patients and the diabetic patients not on metformin (*P* = 0.044), otherwise the differences were not significant.

**Table 1:** Descriptive statistics of diffuse large B-cell lymphoma with or without diabetes type 1/2 treated with rituximab-doxorubicin based chemotherapy at Roswell Park Comprehensive Cancer Center

	All Patients	Non-Diabetic	Diabetic No Metformin	Diabetic Metformin
**Number (%)**	264	215 (81.4%)	21/49 (8.0%)	28/49 (10.6%)
**Median Age**	60	58	73	63.5
**Sex**				
**F/M**	92/172 (34.8%/65.2%)	73/142 (34.0%/66%)	6/15 (28.6%/71.4%)	13/15 (46.4%/53.6)
**Subtype**				
**GCB**	70 (26.5%)	55 (25.6%)	6 (28.6%)	9 (32.1%)
**Non-GCB**	94 (35.6%)	76 (35.3%)	8 (38.1%)	10 (35.7%)
**UNK**	100 (37.9%)	84 (39.1%)	7 (33.3%)	9 (32.1%)
**Stage**				
**I-II/III-IV**	94/167 (36.0%/64.0%)	79/134 (37.1%/62.9%)	7/14 (33.3%/66.6%)	8/20 (28.6%/71.4%)
**IPI Risk Category**				
**High**	19 (7.2%)	16 (7.4%)	1 (4.8%)	2 (7.1%)
**High-Int**	58 (22.0%)	41 (19.1%)	8 (38.1%)	9 (32.1%)
**Low-Int**	97 (36.7%)	78 (36.3%)	8 (38.1%)	11 (39.3%)
**Low**	90 (34.1%)	80 (37.2%)	4 (19.0%)	6 (21.4%)
**Treatment Type**				
**R-CHOP**	231 (87.5%)	185 (86.0%)	19 (90.5%)	27 (96.4%)
**R-CHOP-Doxil**	8 (3.0%)	6 (2.8%)	2 (9.5%)	0 (0%)
**R-CHOP-MTX**	2 (0.8%)	2 (0.9%)	0 (0%)	0 (0%)
**R-EPOCH**	23 (8.7%)	22 (10.2%)	0 (0%)	1 (3.6%)
**Radiation Not treated vs. Treated**	182/78 (70.0%/30.0%)	145/67 (68.4%/31.6%)	18/2 (90.0%/10.0%)	19/9 (67.9%/32.1%)

From December 1997 through May 2013, 264 patients with DLBCL were treated with R-CHOP/R-CHOP-like or R-DA-EPOCH at RPCCC. These patients were identified through analysis of the RPCCC lymphoma database. Their demographic, clinical, and pathological data were compared. The following variables were measured and compared: mean age, sex, DLBCL subtype, stage at diagnosis, IPI risk category, treatment type, and whether or not they received radiation

### Metformin use among diabetic patients associated with improved PFS and OS

**Fig. 1:**
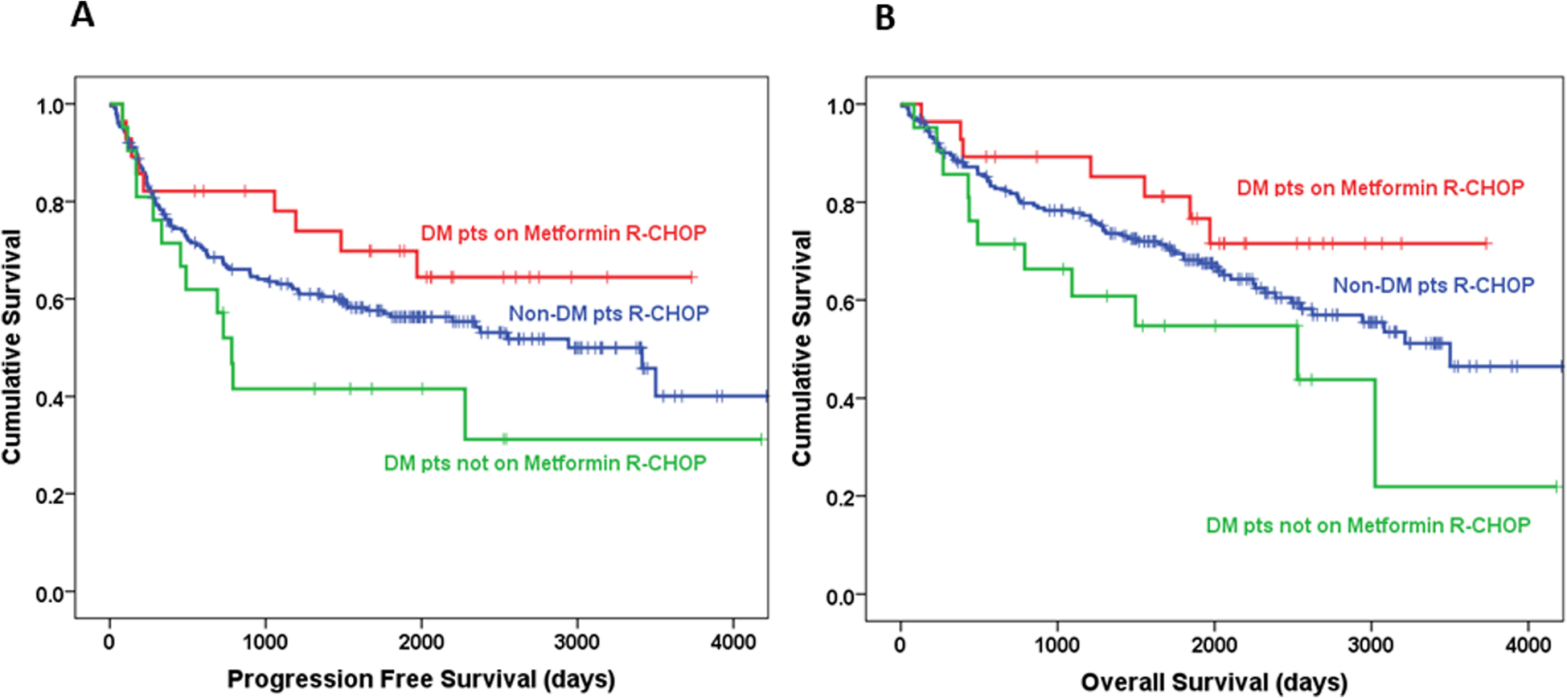
Effects of the use metformin during first-line chemo-immunotherapy in diffuse large B-cell lymphoma patients. Retrospective analysis of 264 patients at RPCCC diagnosed with DLBCL. Kaplan-Meier curves showing an improved progression-free survival (PFS) (**a**) and overall survival (OS) (**b**) based on metformin use in diabetic DLBCL patients. When the impact of metformin use during rituximab and systemic chemotherapy (doxorubicin-based poly-chemotherapy) was compared among all patients, diabetic patients on metformin (red line) vs. other glucose lowering agents (green line) had a statistically significant improvement in both PFS (90 months vs. 60 months, *P* = 0.036) and OS (100 months vs. 71 months, *P* = 0.039). There was a trend towards improved PFS (90 months vs. 83 months, *P* = 0.23) and OS (100 months vs 97 months, *P* = 0.25) even when compared to non-diabetic patients (blue line), but this difference did not reach statistical significance

The use of metformin in DLBCL patients treated with front-line chemo-immunotherapy resulted in improved clinical outcomes (Fig. 1a, b). Among diabetic patients on metformin vs. diabetic patients on other glucose lowering agents, there was a statistically significant improvement in both PFS (90 months vs. 60 months, *P* = 0.036) and OS (100 months vs. 71 months, *P* = 0.039). There was a trend towards improved PFS (90 months vs. 83 months, *P* = 0.23) and OS (100 months vs. 97 months, *P* = 0.25) even when comparing DM patients on metformin to non-diabetic patients, but this difference did not reach statistical significance.

### Metformin single agent results in time and dose-dependent killing and decreased cellular proliferation

**Fig. 2:**
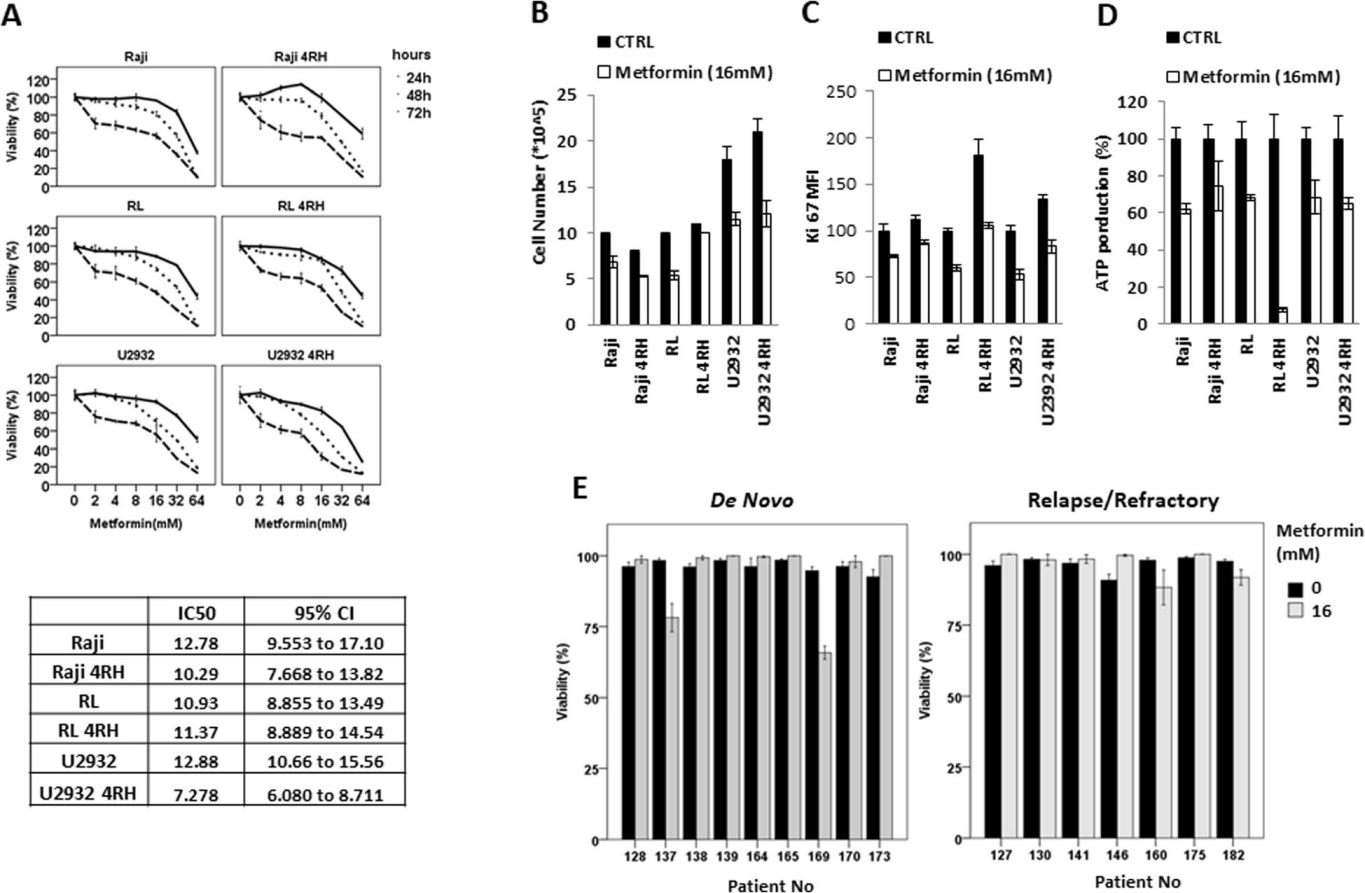
Metformin inhibited rituximab-sensitive and resistant B-cell lymphoma cell lines viability. **a** RSCL and RRCL were treated with accumulative concentration of metformin for 24, 48, or 72 h, respectively. Presto blue assays were performed to assess growth inhibition of metformin. The results are determined as percentage of viable cells compared with control. IC_50_ results were obtained from GraphPad Prism6 after metformin 72 h treatment. **b** RSCL and RRCL cells were cultured in the presence or absence of metformin 16 mM for 48 h. Cell number in each condition was counted by Trypan blue. **c** Ki-67 was stained followed by flow cytometry analysis in RSCL and RRCL exposed to DMSO control or metformin (16 mM) for 48 h. **d** Cells were exposed to metformin 16 mM for 48 hours, and then ATP levels in each of cell lines were assessed by Titer Glo and normalized to the cell number. ATP production was calculated as percentage to the untreated control (100%). **e** Primary tumor cells isolated from patients with either newly diagnosed or relapsed/refractory B-cell lymphoma cells were exposed to metformin (16 mM) for 48 h. Titer Glo assay was performed to assess growth inhibition. The results are determined as percentage of viable cells compared with control. Data represented mean ± SD derived from 3 independent experiments

The addition of metformin to RSCL and RRCL resulted in a dose and time-dependent decrease in cell viability (Fig. 2a). Metformin was also directly cytotoxic to cells, resulting in decreased cell number (Fig. 2b) and decreased proliferative index (Ki-67 index), in both RSCL and RRCL (Fig. 2c). The exposure of metformin resulted in a reduction of ATP (Fig. 2d) in RSCL and RRCL. We explored if metformin induced cell death in primary tumor cells isolated from lymphoma patients (*N* = 16, Supplemental Table 1). To lesser degree when compared to cell lines, activity was seen in samples isolated from de novo or relapsed/refractory lymphoma patients. Differences in the proliferation rate between primary tumor cells and established lymphoma cell lines could explain this finding (Fig. 2e).

### Metformin induces apoptosis, increased ROS production, and lowered mitochondrial potential

**Fig. 3:**
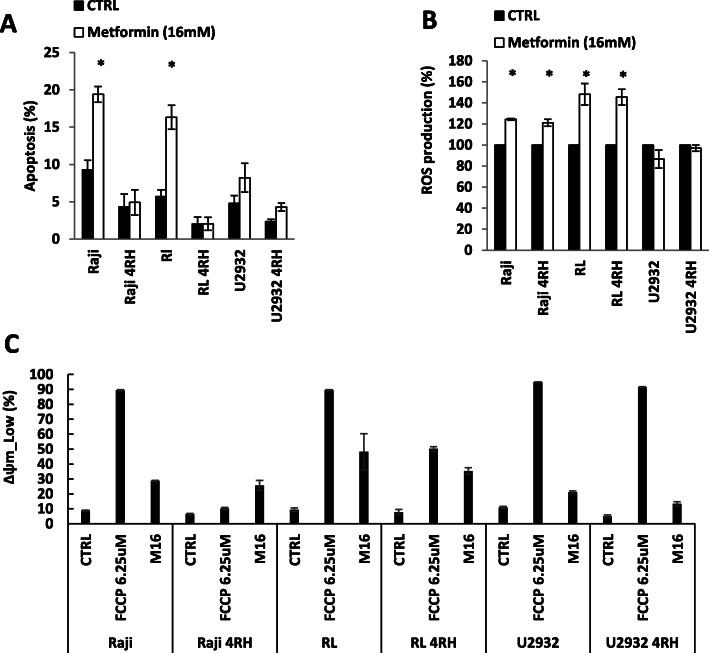
Metformin induced apoptosis, increased ROS production, and decreased the mitochondrial potential in vitro. **a** RSCL and RRCL were exposed to control or metformin (16 mM) for 48 h, and then the percentage of cells undergoing apoptosis (Annexin V + cells) was determined by flow cytometry. Asterisk indicates that the differences were significant at *P* < 0.05. **b** Oxidative stress induced by metformin was determined by comparing dihydrorhodamine-123 (DHR-123) fluorescence intensity at 24 h post-metformin exposure to DHR-123 fluorescence of controls. All data were normalized to the control cell as 100% ROS production. Data represented mean ± SD derived from 3 independent experiments. Asterisk means *P* < 0.05. **c** RSCL and RRCL were cultured for 48 h in the absence or presence of metformin (16 mM). Subsequently, cells were stained for 30 min at 37 °C with DiOC6 followed by flow cytometry analysis. Low DiOC6 population was gated for low mitochondrial outer membrane potential (∆Ψm-Low) and FCCP was used as a positive control. All data represented mean ± SD derived from 3 independent experiments

Mitochondria are the energy factory of the cell, but also regulate apoptosis. One of the key events in apoptosis is the loss of the mitochondrial membrane potential. The loss of the membrane potential results in caspase activation, the loss of mitochondrial functions essential for cell survival as well as the release of molecules that are involved in caspase-independent cell death [32–34]. Once there has been a loss of the mitochondrial membrane potential, the cell has committed to undergo apoptosis. We sought to identify the effect of metformin on apoptosis, ROS production, and mitochondrial membrane potential. The exposure of RSCL and RRCL to metformin resulted in an increase in apoptosis, measured by the percentage of Annexin V positive cells (Fig. 3a). While apoptosis was noted in both RSCL as well as RRCL, the effect of metformin was more pronounced in RSCL.

Subsequently, we examined the effect of metformin on the production of ROS (Fig. 3b). In both Raji and RL cell lines (RSCL and RRCL), metformin resulted in an increase in ROS production. Unlike the Raji and RL cell clines, the U2392 cell lines did not show an increase in ROS production when exposed to metformin. Lastly, we examined the effect of metformin on the mitochondrial membrane potential (Fig. 3c). The loss in mitochondrial membrane potential was more pronounced in RSCL although it was seen in both RSCL and RRCL.

### Metformin induces G1 cell cycle arrest in both RSCL and RRCL

**Fig. 4:**
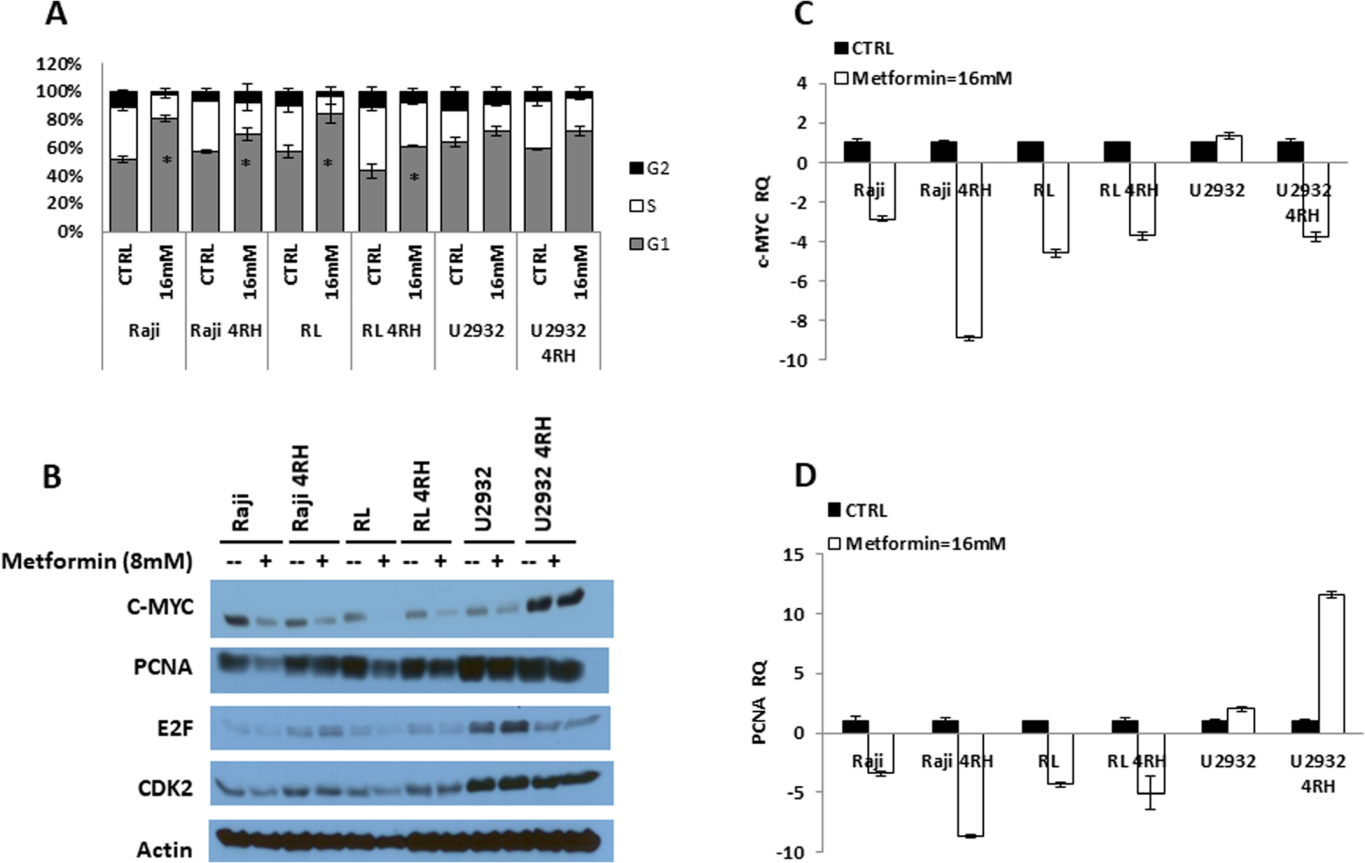
Metformin inhibited G1 cell cycle regulators and induces G1 cell cycle arrest in both RSCL and RRCL. **a** RSCL and RRCL were exposed to DMSO or metformin (16 mM) for 48 h, and then cell cycle distribution changes was determined by flow cytometry. Cumulative results of G1/S/G2-M phases are shown as mean ± standard error of the mean (SEM) (*n* = 3). Column, results are mean + SD of 3 independent experiments. Asterisk means *P* < 0.05. **b** Effect of metformin on cell cycle proteins. Western blot of G1 cell cycle regulatory proteins levels in metformin-induced RSCL and RRCL. **c**, **d** Effects of metformin on transcriptional levels of C-Myc and PCNA. Cells were treated with metformin 16 mM for 24 h. C-Myc and PCNA mRNA levels were analyzed by real-time qPCR

Prior studies [35–37] had shown that in vitro addition of metformin to solid tumor cancer cells resulted in cell cycle arrest. To further investigate the effect of metformin on the cell cycle in lymphoma cells, we performed flow cytometric analysis of cells in response to the treatment with metformin for 48 h. In both RSCL and RRCL, metformin exposure led to cell cycle arrested in the G1 phase and a decrease in the S phase (Fig. 4a). This effect was seen in all cell lines and among RSCL as well as RRCL; however, the effect was the greatest among the Raji and RL (GCB) cell lines compared to U2392 (non-GCB). To further characterize how metformin affects the cell cycle distribution in lymphoma cells, we study changes in G1 cell cycle regulatory proteins following drug exposure (Fig. 4b). In Raji and RL cells, in vitro exposure to metformin resulted in a decrease in C-Myc, PCNA, E2F, and CDK2 as detected by Western blotting. We further explored the mRNA changes of C-Myc and PCNA after exposure to metformin (Fig. 4c, d). Exposure to metformin resulted in a marked decrease in the mRNA levels of C-Myc and PCNA in the Raji and RL cell lines consistent with what was observed in the Western blots.

### Metformin enhances cytotoxic killing by chemotherapy and rituximab’s activity in vitro and in vivo

**Fig. 5:**
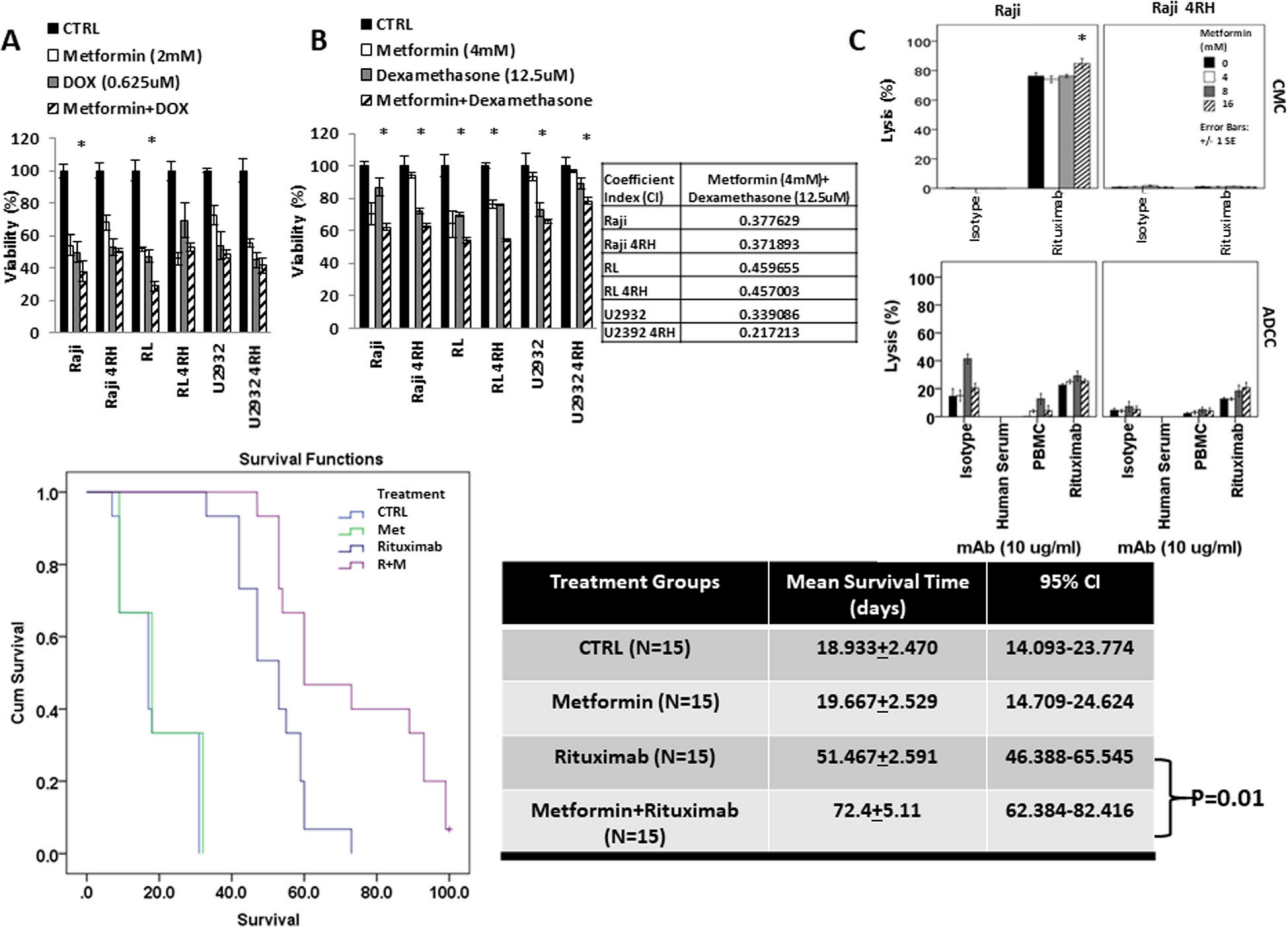
Metformin enhanced chemotherapy and rituximab’s activity in vitro and in vivo. **a**, **b** Metformin enhances doxorubicin and dexamethasone anti-tumor killing activity in RSCL and RRCL. RSCL and RRCL were exposed to metformin for 48 h and changes in cell viability assessed by the change in Presto blue reduction as measured by flow cytometry. Data represented mean ± SD derived from 3 independent experiments. Asterisk means *P* < 0.05. **c** Metformin enhanced anti-CD20 antibody-mediated complement-mediated cytotoxicity (CMC) and antibody-dependent cellular cytotoxicity (ADCC) in RSCL and RRCL. Cells were exposed to media or metformin for 24 h and subsequently labeled with ^51^Cr. Labeled cells were then exposed to isotype or anti-CD20 antibodies, and 20% human serum pooled from healthy volunteers (CMC) or peripheral blood mononuclear cells at an effector:target ratio of 40:1 (ADCC) and incubated at 37 °C, 5% CO_2_ for 6 h. ^51^Cr release was measured and the percentage of lysis was calculated. Asterisk means *P* < 0.05. **d** Metformin potentiated the anti-tumor activity of rituximab in mouse. Survival differences between groups were compared using log-rank analysis. Experiments were repeated in three separate times. The survival difference between rituximab as a single agent compared to rituximab combined with metformin at 2 μg/ml dosage was found to be significant (*P* = 0.01)

To examine if metformin could enhance the anti-tumor activity of chemotherapy drugs, we next tested cell viability with RSCL and RRCL after exposure to metformin and/or chemotherapy agents. Synergistic activity was observed when metformin was combined with doxorubicin (Fig. 5a) in RSCLs (the combination index (CI) of Raji is 0.7 and CI of RL is 0.8). There was also a trend towards improvement in the U2392 and the RRCL, but these did not reach statistical significance. The addition of metformin to dexamethasone (Fig. 5b) resulted in a statistically significant decrease in cell viability in all cell lines tested except the U2392 cell line. The CI value calculated by the CalcuSyn software was less than 0.5 (Fig. 5c), which indicated a synergistic effect between metformin and dexamethasone among our RSCL and RRCL.

We then tested to see if metformin could enhance the activity of rituximab by exposing RSCL and RRCL to varying concentrations of metformin and then evaluated complement-mediated cytotoxicity (CMC) and antibody-dependent cellular cytotoxicity (ADCC) in the presence of rituximab (Fig. 5c). RSCL showed increased CMC in a dose-dependent manner to metformin. There was no effect on CMC in RRCL. The addition of metformin with rituximab resulted in increased ADCC in both RSCL and RRCL.

The treatment of human lymphoma-bearing SCID mice with rituximab resulted in prolongation in survival as compared with placebo-treated controls (Fig. 5d). The administration of metformin in combination with rituximab resulted in the most effective anti-tumor activity and prolongation of survival of human lymphoma-bearing SCID mice. Statistically significant differences were observed between animals treated with rituximab vs. metformin plus rituximab. The median survival time of animals treated with metformin and rituximab was longer (72.5 ± 5.11 days) than those treated with rituximab monotherapy alone (median survival of 51.5 ± 2.6 days); log-rank test, *P* = 0.01.

## Discussion

**Fig. 6:**
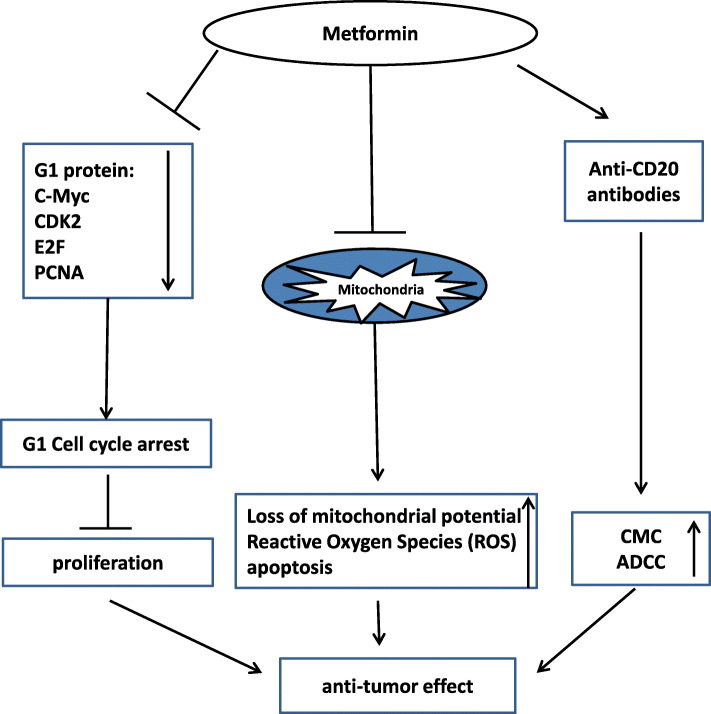
Proposed model of anti-tumor mechanism for metformin in rituximab-sensitive and resistant B-cell lymphoma. Based on our work, we have formulated a model by which we hypothesize metformin induces in its anti-tumor effects. Metformin inhibits proliferation via C-Myc resulting in G1 cell cycle arrest. Metformin causes increased ROS production and loss of mitochondrial membrane potential resulting in apoptosis. Metformin enhances rituximab-mediated ADCC and to a lesser degree CMC

Our contribution highlights the effect of metformin in aggressive B-cell lymphoma starting with a retrospective analysis in DLBCL patients, and then working backwards using pre-clinical models. This study is relevant for future drug design, as well as clinical study design for patients with DLBCL, particularly in underdeveloped countries where access to costly treatments are an ongoing challenge. Our initial research was a retrospective analysis of a large cohort of patients with DLBCL at a single institution where we found that the use of metformin was associated with improved clinical outcomes. We then performed studies on pre-clinical models where we showed that metformin acts via several mechanisms to both exert anti-tumor effects independently, as well as to enhance the anti-tumor effects of traditional chemotherapy. We identified E2F as a potential target of metformin that has not been previously identified. Based on our research, we hypothesize a model for the anti-tumor mechanism for metformin (Fig. 6).

In pre-clinical modes, we saw that metformin as a single agent was able to affect time and dose-dependent killing in RSCL as well as RRCL. This was shown in both cell lines as well as in patient-derived primary cells. The time and dose-dependent response were found at metformin IC_50_ of 7–13 mM. The metformin drug concentrations utilized in our in vitro experiments are higher than the estimated drug concentrations achieved in our in vivo experiments. These discrepancies were also noted in other scientific contributions. The higher dose of metformin needed for in vitro experiments could be partially due to the media used during the experimental design. As described by Heiden et al., pyruvate, glucose, and aspartate which are common ingredients in culture media (i.e., DMEM and RPMI1640) can diminish metformin sensitivity [38]. Another second possible explanation to explain the higher doses of metformin required in in vitro experiments, in our rituximab-chemotherapy-resistant cell lines, is their known abnormal metabolic state and apoptotic threshold [7].

Cellular proliferation, as measured by Ki-67, has been associated with worse outcomes in DLBCL [39]. In all cell lines, the exposure to metformin resulted in a decrease in the Ki-67. Metformin’s effects on mitochondria are complex, but one of its mechanisms is the inhibition of complex I of the electron transport chain [37, 40]. Prior studies in breast cancer models have shown that metformin causes a decrease in ATP production, which subsequently leads to a decrease in cellular proliferation [37]. Cancer cells typically shift their metabolism to anaerobic glycolysis to help them produce ATP even in anaerobic environments (Warburg’s effect) [17, 41]. Research in breast cancer models suggested that cancer cells that were more dependent on glycolysis were in fact more susceptible to inhibition of the complex I, while there was reduced or absent effect on normal cells [41]. Our research shows that in DLBCL RSCL as well as RRCL, the addition of metformin resulted in a decrease in ATP production which may be responsible for the decreased cellular proliferation seen in RSCL and RRCL treated with metformin.

The effect of metformin on cellular proliferation has been shown in several other malignant cell lines [42]. Our findings are consistent with other investigators. The mechanisms by which metformin affects cellular proliferation in DLBCL are multifactorial, but we have shown that arrest of the cell in the G1 phase, due to downregulation of E2F, as well as decreased ATP production is both caused by metformin, and result in decreased cellular proliferation.

Metformin has been shown to induce apoptosis in leukemia, breast, and esophageal cancer cell lines [43–45]. We show here that exposure to metformin resulted in dose-dependent increase in apoptosis in both RSCL and RRCL. Prior work by our group has shown that the development of RRCL is associated with a decrease in the pro-apoptotic proteins Bax and/or Bak [4]. These two proteins are responsible for oligomerization and loss of the mitochondrial membrane potential. The findings from Fig. 3a are thus consistent with prior work, showing that while metformin results in increased apoptosis, this effect is less pronounced in RRCL, and this may be due to the decreased levels of Bax and/or Bak in these cell lines. Interestingly, even in the RRCL, metformin was able to cause an increase in apoptosis.

It has been shown previously that excessive mitochondrial oxidant stress can induce cell death in tumors through cytochrome release and apoptosis [36, 46]. We show here that addition of metformin results in an increase in ROS and loss of the mitochondrial membrane permeability in lymphoma cells. Together, these suggest that the increase in apoptosis is mediated by ROS production resulting in increased membrane permeability. There is likely another factor(s) causing the loss in mitochondrial membrane potential besides increased ROS production, as there was essentially no increase in ROS production in the U2392 cell lines on exposure to metformin.

Cell cycle arrest can affect cancer cell proliferation, besides induction of apoptosis. The transition from G1 to S phase is controlled by several growth-dependent cyclin-dependent kinases (CDK) and cyclins which will complex to guide the cell through the cycle. In the early G1 phase, mitogenic stimulation results in synthesis and formation of cyclin D/CDK4/6 complexes. This causes phosphorylation of the RB protein (and their family of proteins). This is a so-called “restriction point,” and after this phosphorylation, the cell has committed to progression through from G1 to S phase. Once it is phosphorylated, pRB dissociates from E2F. This dissociation allows E2F to allow expression of genes for DNA synthesis [47–49]. Metformin’s effects on the cell cycle distribution have been shown previously [35–37, 50]. Here, we have also demonstrated that metformin induces G1 cell cycle arrest, with a decrease in the cellular proteins C-Myc, CDK2, E2F, and PCNA. This is confirmed by the decrease in mRNA of C-Myc and PCNA, both of which correspond to the S Phase [51–53]. Of note, our data show that although there was arrest of the cell cycle in G1 for U2392 cell lines, there was minimal effect on C-Myc, PCNA, E2F, or CDK2. The U2392 cell line is derived from an ABC DLBCL cell line as opposed to the Raji and RL cell lines, which are derived from GCB DLBCL and BL respectively. It is possible that mechanism of cell cycle arrest may be different in the ABC cell lines as opposed to GCB-DLBCL or BL cell lines.

## Conclusion

As metformin is becoming more studied for its anti-tumor effects, numerous pre-clinical and clinical studies have also begun focusing on combining metformin with chemotherapy in solid tumor malignancies [19]. Mouse models have shown that metformin plus chemotherapy is more effective than either agent alone [54–56]. We are one of the first groups to report synergistic activity when metformin is combined with either doxorubicin or dexamethasone in lymphoma pre-clinical models. This finding supports the observation noted in our retrospective analysis of DLBCL patients receiving first-line chemotherapy at our Institute. In addition, the cellular effects of metformin can re-sensitize chemotherapy resistant cells to chemo- and immunotherapy drugs. We are the first to demonstrate here that metformin enhances ADCC of Rituximab in vitro. This effect was seen in both RSCL as well as RRCL and was dose-dependent. To confirm this effect, we studied the effect of adding metformin to rituximab in a lymphoma SCID mice model. Here, we showed that metformin plus rituximab was synergistic in improving the survival of lymphoma bearing SCID mice. This aids in supporting the evaluation of combining metformin with rituximab and/or chemotherapy agents in aggressive B-cell lymphoma clinical trials.

Our work does have a notable limitation. The concentration of metformin in our studies was significantly higher than the typical plasma levels of metformin, which are typically on the order of 10–40 μM [40, 57]. However, in real-world scenarios, patients are typically being exposed constantly to metformin for months to years, potentially leading to broader range in drug blood concentrations. Thus, although they are not exposed to similar plasma concentrations, they are exposed to much longer durations of metformin that are not feasible to perform in laboratory models. It also suggests that by understanding how metformin exercises its anti-tumor effects, a novel inhibitor could be developed with a better therapeutic range concentration. Additionally, while our work has shown some single agent activity for metformin, we are not advocating for the use of metformin as a single agent, but rather as chemotherapy enhancer.

## Supplementary information

**Additional file 1: Supplemental Table 1.** Descriptive statistics of 16 patient samples treated at Roswell Park Comprehensive Cancer Center. Primary neoplastic B-cells were isolated from pre-treatment biopsy tissue obtained from 16 patients with previously untreated (N=9) or relapsed/refractory (N=7) B-cell NHL receiving therapy at Roswell Park Comprehensive Cancer Center (RPCCC). Samples from patient biopsy specimens were procured under Institutional Review Board (IRB) RPCCC protocols I42804 and I42904. Tissue specimens were placed in PBS-containing collagenase type IV (1mg/ml; Sigma-Aldrich, St. Louis, MO) and incubated for 15 minutes at 37°C, followed by manual agitation for five minutes. Next, samples were diluted with RPMI 1640-containing 10% fetal bovine serum (FBS) and the cell suspension filtered through a 100μm cell strainer to remove large clumps. Lymphocytes were enriched by density centrifugation. B-cells were then isolated from enriched lymphocytes by MACS separation using a human B-cell Isolation Kit II (Miltenyi Biotec, Gladbach, Germany).

## Data Availability

All data generated or analyzed during this study are included in this published article and its supplementary information files.

## Abbreviations

DLBCL: Diffuse large B-cell lymphoma
RSCL: Rituximab-sensitive cell lines
RRCL: Rituximab-resistant cell lines
ROS: Reactive oxygen species
