# Supplementary material for: Metformin sensitizes therapeutic agents and improves outcome in pre-clinical and clinical diffuse large B-cell lymphoma
Source: Cancer Metab. 2020 Jul 6;8:10. doi: 10.1186/s40170-020-00213-w (PMC7336499; doi:10.1186/s40170-020-00213-w)
Supplement: Supplementary file 1 — Additional file 1: Supplemental Table 1. Descriptive statistics of 16 patient samples treated at Roswell Park Comprehensive Cancer Center. Primary neoplastic B-cells were isolated from pre-treatment biopsy tissue obtained from 16 patients with previously untreated (N=9) or relapsed/refractory (N=7) B-cell NHL receiving therapy at Roswell Park Comprehensive Cancer Center (RPCCC). Samples from patient biopsy specimens were procured under Institutional Review Board (IRB) RPCCC protocols I42804 and I42904. Tissue specimens were placed in PBS-containing collagenase type IV (1mg/ml; Sigma-Aldrich, St. Louis, MO) and incubated for 15 minutes at 37°C, followed by manual agitation for five minutes. Next, samples were diluted with RPMI 1640-containing 10% fetal bovine serum (FBS) and the cell suspension filtered through a 100μm cell strainer to remove large clumps. Lymphocytes were enriched by density centrifugation. B-cells were then isolated from enriched lymphocytes by MACS separation using a human B-cell Isolation Kit II (Miltenyi Biotec, Gladbach, Germany). [file 40170_2020_213_MOESM1_ESM.pdf]

Descriptive statistics of 16 patient samples treated at Roswell Park Comprehensive Cancer Center

	All Patients	De Novo	Relapsed/Refractory
Number (%)	16	9	7
Mean Age	61.7	60.9	62.7
Sex<br>F/M	5/11<br>(31.2%/68.8)	3/6<br>(33.3%/66.7%)	2/5<br>(28.6%/71.4%)
Subtype<br>Mantle Cell	5 (31.3%)	4 (44.4%)	1 (14.3%)
Marginal Zone	8 (50.0%)	5 (55.6%)	3 (42.9%)
FL	2 (12.5%)	0 (0%)	2 (28.6%)
DLBCL/ABC	1 (6.3%)	0 (0%)	1 (14.3%)

Supplemental Table 1
